# Surgical management of Sternal wound infection post cardiac surgery "single surgeon experience"

**DOI:** 10.1186/1749-8090-10-S1-A275

**Published:** 2015-12-16

**Authors:** Hamidreza Davari, Nahid Hassanpour, Peyvand Bina

**Affiliations:** 1 Tehran Heart Center, Tehran University of Medical Sciences, Tehran, 14117113137, Iran

## Aims/Objective

Sternal osteomyelitis and post sternotomy mediastinitis still stays as a severe life-threatening complication after cardiac surgery. This study was conducted to evaluate the short and long term results of surgical management of deep sternal wounds infection (DSWI).

## Methods

A retrospective study was conducted to investigate post cardiac surgery patients with sternal wound infection by thoracic surgeon. From March 2008 to March 2013 all patients who were underwent surgery for post cardiac surgery sternal wound infection and or dehiscence were enrolled.

## Results

 A consultant non cardiac thoracic surgeon managed 146 patients with DSWI and /or dehiscence. Nine patients were excluded due to sternal dehiscence with no evidence of gross infection and also negative wound culture. Eighty four patients were female. According to El Oakley classification, Type I, II, III, IV, V were 8, 2, 79,5,43, consequently. All patients evaluated by CT scan before intervention or after 1st operation. Patients had soft tissue debridement, with removal of wires in one or staged operation combined with mediastinotomy, mediastinal irrigation and debridement. Twelve patients had near total sternectomy, one upper sternectomy and one patient had sternoclavicular joint resection. One hundred four patient had partial sternectomy (74 longitudinal and 30 transverse sternectomy. Chondrectomy was done in 44 and decortication in 6. Twelve patient had rewiring. Bone stabilization was done by Zipfix or Sternal band +/_ wires in 12 and Titanium plate in 6.Only two patients had negative pressure wound therapy (NPWT) as a bridge to reconstruction. At the end 20 patients had delay simple closure of skin. In 113 patients a total of 163 pectoral muscle flap including: 36 right pectoral major muscle turnover flap (RPMMTF); 53 combined RPMMTF plus left advancement pectoral muscle flap (LTPMMFC); 14 local right or left PMMF were done. We used 4 rectus abdominis flap, 2 omental flap, and breast flap. Twenty nine patients had reoperation after reconstruction. Nine had recurrence osteomyelitis and /or chondritis. One patient had mediastinitis and 9 had skin flap necrosis. Remnant of pace wire was the cause of recurrent infection in 2. Five patients had rewiring or reconstruction and one patient had operation due to GI bleeding. Hospital mortality rate was 8.21% (12 patients were died. Patients had followed for a mean of 40.3 months and 3 years mortality rate was 13.69%.

## Discussion/Conclusions

DSWI remains a major challenging complication after open heart surgery. The key issues are early diagnosis, prompt surgical intervention by expertise and use all available treatment modality to decrease mortality and better outcomes.

